# Supplementary material for: Mapping the evolution of fertility support policies in China: A content and instrumental analysis
Source: PLoS One. 2025 Oct 9;20(10):e0332137. doi: 10.1371/journal.pone.0332137 (PMC12510515; doi:10.1371/journal.pone.0332137)
Supplement: S1 Appendix — (ZIP) [file pone.0332137.s001.zip › S1 Appendix. 226 original policy documents/182-国家发展和改革委员会关于黔中经济区发展规划的批复(FBM-CLI-4-201159).doc]

国家发展和改革委员会关于黔中经济区发展规划的批复

**发布部门：** [国家发展和改革委员会(含原国家发展计划委员会、原国家计划委员会)](https://www.pkulaw.com/law/chl?Aggs.IssueDepartment=60301) [机构沿革](javascript:void(0))

**发文字号：**发改西部〔2012〕2446号

**发布日期：**2012.08.12

**实施日期：**2012.08.12

**时效性：** [现行有效](https://www.pkulaw.com/law/chl?Aggs.TimelinessDic=01)

**效力级别：** [行政许可批复](https://www.pkulaw.com/law/chl?Aggs.EffectivenessDic=XE0304)

**法规类别：** [区域经济开发](https://www.pkulaw.com/law/chl?Aggs.Category=02308)

国家发展和改革委员会关于黔中经济区发展规划的批复
（发改西部[2012]2446号）

贵州省人民政府：
　　报来《贵州省人民政府关于请求审批＜黔中经济区发展规划＞的请示》（黔府函[2011]283号）收悉。经商国务院有关部门，现批复如下：
　　一、原则同意《黔中经济区发展规划》（以下简称《规划》），请认真做好组织实施工作。

　　二、《规划》实施要高举中国特色社会主义伟大旗帜，以邓小平理论和“三个代表”重要思想为指导，深入贯彻落实科学发展观，以加速发展、加快转型、推动跨越为主基调，全面实施工业强区和城镇化带动战略，着力加强基础设施和生态环境建设，提升发展保障能力；着力推进工业化信息化融合，构建现代产业体系；着力优化区域发展格局，带动周边地区共同发展；着力保障和改善民生，推进基本公共服务均等化；着力深化改革扩大开放，推进体制机制创新，将黔中经济区培育成为高科技、优产业、开放型、生态化、经济快速崛起、城乡协调发展的核心区域，走符合自身实际和时代要求的后发赶超之路，为贵州省实现全面建设小康社会宏伟目标奠定坚实基础。

　　三、请切实加强对《规划》实施的组织领导，明确分工，落实责任，完善机制，制定具体实施方案，确保《规划》提出的战略布局、重点任务、政策措施落实到位，按时完成发展目标。要加强与国务院有关部门的衔接，《规划》中涉及的政策措施和建设项目需按程序另行报批。要督促《规划》范围内的各市（州）人民政府加强沟通协调，打破行政区划，促进要素流通，形成区域发展合力。

　　四、国务院西部地区开发领导小组成员单位及有关部门要加强对黔中经济区发展的指导、协调和支持。我委将会同有关部门和你省加强对《规划》实施情况的跟踪分析，重大问题要及时向国务院报告。
　　请你省以《规划》实施为契机，抢抓机遇，开拓创新，密切配合，狠抓落实，推动贵州与全国同步实现建成小康社会宏伟目标。
　　附件：黔中经济区发展规划

国家发展改革委
2012年8月12日

　　附件：

黔中经济区发展规划
〇二一二年八月
目　 录

　　前　言
　　第一章　发展基础
　　第一节　发展优势
　　第二节　机遇与挑战
　　第三节　重要意义
　　第二章　总体要求
　　第四节　指导思想
　　第五节　战略定位
　　第六节　发展目标
　　第三章　空间布局
　　第七节　贵阳安顺一体化核心区
　　第八节　三带多中心
　　第九节　辐射带动区域
　　第四章　新型工业化
　　第十节　能源资源深加工业
　　第十一节　装备制造业
　　第十二节　战略性新兴产业
　　第十三节　特色轻工业
　　第十四节　循环经济
　　第五章　现代服务业
　　第十五节　文化产业
　　第十六节　旅游业
　　第十七节　商贸物流业
　　第十八节　金融及其他服务业
　　第六章　新型城镇化
　　第十九节　城市发展
　　第二十节　贵安新区
　　第二十一节　重点城镇
　　第二十二节　市政基础设施
　　第二十三节　城乡统筹发展
　　第七章　基础设施
　　第二十四节　交通运输
　　第二十五节　水利设施
　　第二十六节　能源通道
　　第二十七节　信息
　　第八章　农业与生态环境
　　第二十八节　特色优势农业
　　第二十九节　生态综合治理
　　第三十节　加强环境保护
　　第九章　内陆开放高地
　　第三十一节　对内对外开放
　　第三十二节　完善基础平台
　　第三十三节　承接产业转移
　　第十章　社会事业
　　第三十四节　教育
　　第三十五节　科技
　　第三十六节　公共文化
　　第三十七节　医疗卫生
　　第三十八节　就业和社会保障
　　第十一章　深化改革
　　第三十九节　行政体制改革
　　第四十节　发展非公有制经济
　　第四十一节　创新土地矿产资源开发利用机制
　　第四十二节　创新人才开发机制
　　第十二章　规划实施

　　前　言

　　黔中经济区是新一轮西部大开发战略确定的重点经济区，是以《全国主体功能区规划》明确的重点开发区域为核心、延及周边的经济区域。范围包括贵州省贵阳市及遵义市红花岗区、汇川区、遵义县、绥阳县、仁怀市，安顺市西秀区、平坝县、普定县、镇宁县，毕节市七星关区、大方县、黔西县、金沙县、织金县，黔东南州凯里市、麻江县，黔南州都匀市、福泉市、贵定县、瓮安县、长顺县、龙里县、惠水县，共计 33 个县（市、区）。区域国土面积53802平方公里，占贵州省的31%；2011年常住人口1571万，占贵州省的45%。
　　本规划依据《[中华人民共和国国民经济和社会发展第十二个五年规划纲要](https://www.pkulaw.com/chl/603c7a7f2158b52bbdfb.html)》、《中共中央国务院关于深入实施西部大开发战略的若干意见》、《[国务院关于进一步促进贵州经济社会又好又快发展的若干意见](https://www.pkulaw.com/chl/04822edc315ed85bbdfb.html)》和《全国主体功能区规划》、《[西部大开发“十二五”规划](https://www.pkulaw.com/chl/ecdf6240f906a438bdfb.html)》编制，是指导黔中经济区未来一个时期发展改革的纲领性文件和编制相关专项规划的依据。规划期为 2012-2020年。

　第一章　发展基础

改革开放特别是实施西部大开发战略以来，黔中经济区经济社会发展取得显著成就，站在了新的历史起点上，既面临实现率先发展新的重大历史机遇，也面临诸多不容忽视的挑战。

　第一节　发展优势

区位优势突出。黔中经济区地处西南地区腹心地带，在全国“两横三纵”城市化战略格局中位于包昆通道纵轴南部，区域内贵广铁路、贵广高速公路、长昆客运专线、渝黔铁路等交通大动脉纵横交错，是西南地区重要的陆路交通枢纽。
　　能矿资源富集。水能资源、煤炭资源丰富，煤炭查明储量占西南地区的36%，水煤结合、水火互济的能源组合优势明显。磷、铝、稀土、锰等矿产资源丰富，磷矿资源查明储量占西南地区的42%，是国家重要的能源基地和资源深加工基地。
　　文化独具特色。民族历史文化资源富集，夜郎文化、水西文化、屯堡文化、阳明文化、沙滩文化以及红色文化等历史文化厚重深远，苗、布依、侗、彝等少数民族文化、原生态文化魅力独特，具有景观多元性、形态原生性、内涵厚重性、气度包容性的鲜明文化特质。
　　综合实力较强。2011年地区生产总值3396亿元，占贵州省的60%；固定资产投资2538亿元，占贵州省的50%。以能源资源深加工、装备制造、食品等轻工业为主的特色优势产业初具规模，交通、水利、电力等基础设施保障能力显著增强，基本具备实现率先跨越发展的基础条件。
　　承载能力良好。区域内基本属于喀斯特低丘缓坡地貌，荒山荒坡等未利用地较多。年降水量 1100-1300 毫米，岩溶地下水开发潜力大。气候类型多样，立体特征明显，生物资源多样性良好；雨热基本同期，是区域特色农产品生产基地。森林覆盖率达42%，人居环境良好。

　第二节　机遇与挑战

发展机遇。国家将深入实施西部大开发战略摆在区域发展总体战略中的优先位置，出台进一步促进贵州经济社会又好又快发展的政策性文件，为黔中经济区率先发展营造了良好发展环境。国家实施扩大内需战略，积极推进加快转变经济发展方式，为黔中经济区加强基础设施建设，实现产业结构调整优化提供了重要支撑。国际产业分工深刻调整，东部地区产业加速升级，向中西部地区产业转移步伐加快，为黔中经济区积极承接国际国内产业转移创造了重大契机。贵州举全省之力把黔中经济区打造成为辐射带动周边地区发展的经济增长极，为黔中经济区率先实现加速发展、加快转型、推动跨越提供了重要保障。
　　困难挑战。一是发展方式相对粗放，产业结构调整压力较大，“赶”与“转”任务繁重。二是水资源丰富与工程性缺水并存，水利、交通、能源等重大基础设施建设滞后。三是城镇化进程有待加快，城镇承载能力需要进一步加强，城乡发展不平衡。四是科技投入不足，人才结构不合理，区域创新环境亟待优化。 总体上看，机遇大于挑战。今后一段时期是黔中经济区率先跨越发展、实现经济起飞的重要战略机遇期。

　第三节　重要意义

新形势下推进黔中经济区率先发展，带动贵州全省特别是民族地区尽快富裕起来，是西部欠发达地区与全国缩小差距的一个重要象征，是国家兴旺发达的一个重要标志。推进黔中经济区率先发展，有利于探索内陆欠发达地区后发赶超、实现跨越的新途径，有利于保障区域能源安全和经济安全，有利于进一步优化国家生产力布局，有利于带动贵州民族地区共同发展，实现贵州与全国同步建成小康社会宏伟目标。

　第二章　总体要求

　第四节　指导思想

高举中国特色社会主义伟大旗帜，以邓小平理论和“三个代表”重要思想为指导，深入贯彻落实科学发展观，以加速发展、加快转型、推动跨越为主基调，全面实施工业强区和城镇化带动战略，着力加强基础设施和生态环境建设，提升发展保障能力；着力推进工业化信息化融合，构建现代产业体系；着力优化区域发展格局，带动周边地区共同发展；着力保障和改善民生，推进基本公共服务均等化；着力深化改革扩大开放，推进体制机制创新，将黔中经济区培育成为高科技、优产业、开放型、生态化、经济快速崛起、城乡协调发展的核心区域，走符合自身实际和时代要求的后发赶超之路，为贵州省实现全面建设小康社会宏伟目标奠定坚实基础。

　第五节　战略定位

国家重要能源资源深加工、特色轻工业基地和西部地区装备制造业、战略性新兴产业基地。充分发挥能矿资源组合良好、生物资源多样性丰富的优势，依托技术创新平台，重点发展煤、磷、有色等资源深加工业，食品、民族医药等特色轻工业，航空航天、能矿机械等装备制造业，生物、新材料等战略性新兴产业，培育特色优势产业集群，构筑现代产业体系，建成西部地区新的经济增长极。
　　国家文化旅游发展创新区。充分发挥自然风光绚丽秀美、四季气候清新宜人、民族文化多姿多彩、红色文化影响深远的优势，探索推动文化与旅游融合发展新路径，推进体制机制改革，创新文化走出去模式，不断解放和发展文化生产力，建设国际知名、国内一流的休闲度假旅游目的地和文化交流的重要平台。 全国山地新型城镇化试验区。充分利用自然山势、水系和喀斯特地形特点，建设土地节约利用、经济文化繁荣、生态环境优美、人与自然和谐的山水田园城镇。推进统筹城乡改革试验先行先试，创新农村土地利用制度，保障农民权益，探索形成山地城镇化发展的新机制、新经验、新模式。
　　东西互动合作示范区。创新区域合作机制、构建对内对外开放平台，以开发区和产业园区为主要载体，推进与珠三角、成渝经济区等区域的经贸合作与交流，建设国内外产业转移的重要承接区和出口商品加工基地，打造富有活力和竞争力的内陆开放新高地。
　　区域性商贸物流中心。充分发挥区位优势，建设跨区域交通通道，推进现代物流服务和信息平台建设，加快构建结构合理、功能完备、高效便捷的现代商贸物流体系，建成西南地区重要的物流枢纽。

　第六节　发展目标

到2015年，黔中经济区率先实现跨越发展的主要目标是：
　　--综合经济实力显著增强。地区生产总值力争达到 6000亿元以上，地方财政收入达到450亿元以上。以生物、新材料等战略性新兴产业为先导，以能源资源深加工、装备制造等为主导的产业集群初步形成，工业化和城镇化带动作用显著增强。创新能力稳步提升，科技研发费用达到地区生产总值的2.0%。
　　--基础设施建设取得突破。率先全面实现区域中心城市快速铁路连接、县县通高等级公路和具备条件的建制村通沥青（水泥）路。交通、水利、能源、通信、城镇等基础设施支撑保障能力明显增强，农村生产生活条件大幅改善。
　　--资源节约环境保护取得明显成效。单位地区生产总值能耗显著降低，主要污染物排放总量控制在国家下达的指标范围内。土地资源利用效率明显提高，矿产资源节约与综合利用水平显著加强。森林覆盖率达到45%以上。
　　--人民生活水平大幅提高。九年义务教育质量显著增强，巩固率达到95%以上。建立健全覆盖城乡居民的基本医疗卫生服务体系和社会保障体系，基本公共服务能力明显提升。城乡居民收入力争实现增长步伐与经济发展同步。城镇化率年均增长2个百分点以上。
　　到2020年，地区生产总值和地方财政收入力争比2015年翻一番，现代产业体系基本形成，经济发展质量和效益明显提高，综合竞争力和辐射带动能力显著增强。城镇集聚人口能力进一步增强，城镇体系更加完善。综合交通运输体系和水利工程体系基本建成，石漠化扩展势头得到根本遏制。人民生活更加富裕，覆盖城乡的社会保障体系基本建成，基本公共服务能力达到全国平均水平，全面建成小康社会。

　第三章　空间布局

按照以线串点、以点带面的空间开发模式，推进形成以贵阳-安顺为核心，遵义、毕节、都匀、凯里等中心城市为支撑，快速交通通道为主轴的“一核三带多中心”空间开发格局，辐射带动周边区域发展。

　第七节　贵阳安顺一体化核心区

统筹推进贵阳、安顺经济一体化发展，建设贵安新区，打造大贵阳经济圈。按照优化配置、同城共享的思路，积极推进公共交通、电信服务、教育医疗、社会保障等重要基础设施和基本公共服务一体化发展，形成支撑贵州、引领黔中的都市核心区。重点发展装备制造、电子信息、生物制药、特色食品等优势产业和战略性新兴产业，打造以航空、汽车及零部件为重点的装备制造业基地和绿色轻工业基地。围绕建设商贸物流中心、生态旅游城市和区域科技、金融服务中心，加快发展商贸物流、会展、金融等现代服务业和文化旅游产业。

　第八节　三带多中心

依托交通干线，打造贵阳-遵义，贵阳-都匀、凯里，贵阳-毕节经济带，提升中心城市承载能力。
　　贵阳-遵义经济带。沿贵遵高速公路、渝黔铁路等交通干线，重点发展资源深加工、装备制造、汽车及零部件、新材料、电子信息、新能源、优质烟酒、生物制药等产业。发挥遵义连接成渝和黔中经济区的枢纽作用，打造遵义特大城市，加快建设仁怀、息烽等中小城市和乌江、扎佐等小城镇。
　　贵阳-都匀、凯里经济带。沿贵广铁路、贵广高速公路、长昆客运专线等交通干线，重点发展磷化工、特色轻工和民族文化、旅游等产业。壮大都匀、凯里中心城市人口和经济规模，推进都匀-麻江-凯里城市组团发展，形成区域性大城市和国际旅游城市，建设盘江、昌明等小城镇。
　　贵阳-毕节经济带。沿贵毕高等级公路、成贵铁路等交通干线，重点发展以火力发电为主的能源工业、以煤化工为重点的资源深加工产业、以能矿机械为主的装备制造业以及旅游业。打造毕节特大城市，建成黔中连接川南滇北的交通枢纽和物流中心，建成国家新型煤化工基地。建设织金、黔西等小城市。

　第九节　辐射带动区域

按照“黔中带动、黔北提升、两翼跨越、协调推进”的原则，充分发挥黔中经济区的辐射带动作用，加强与黔北经济协作区、毕（节）（六盘）水兴（义）能源资源富集区、“三州”（黔东南苗族侗族自治州、黔南布依族苗族自治州、黔西南布依族苗族自治州）民族地区的经济联系，促进要素流动和功能整合，实现优势互补、联动发展。努力将黔北经济协作区建设成为连接成渝经济区和黔中经济区的经济走廊，推进武陵山地区经济协作和扶贫攻坚，将铜仁建设成为贵州东北部区域性中心城市。加强毕水兴能源资源富集区与黔中经济区在资源深度开发和转化的分工合作，将六盘水建设成为贵州西部中心城市和重要交通枢纽，将兴义建设成为黔桂滇交界的区域性大城市和商贸物流中心。依托黔中经济区人才、资金、技术优势，带动“三州”民族地区深入挖掘民族文化、旅游、生态等资源，打造具有国际影响力的原生态民族文化旅游区，推动各族群众实现共同富裕。

　第四章　新型工业化

优化产业布局，强化科技创新，积极推进工业化和信息化融合、工业化和城镇化协调发展，促进产业结构调整和优化升级，打造特色优势产业集群和产业基地。

　第十节　能源资源深加工业

充分发挥能矿资源丰富的比较优势，推动能源与矿产资源开发利用协调发展，建设国家重要能源资源深加工基地。
　　煤炭及电力。加大煤炭基地勘查力度，整合、改造现有煤矿，优先建设大中型煤矿，培育大型煤炭企业集团。积极开展煤矸石、煤泥、煤层气等综合开发利用。稳步扩大以煤电为主的电力装机规模，加快电源点建设，建设一批煤矸石综合利用电厂，发展产业园区和城市热电联产机组。大力推动煤电联营，建设绥阳煤电一体化产业基地，重点打造金沙-黔西-织金-普定和七星关-大方-黔西-清镇煤炭煤电产业带。关停贵阳、金沙等小火电机组，淘汰落后生产能力。在保护生态环境的前提下，深度开发水电，稳步推进乌江等重点流域水电开发。发挥水火互济优势，加强发电与用电产业配套，积极推动发电企业与电力用户直接交易。

专栏1：煤炭开发及煤电一体化重点工程

煤矿：新建小屯（120万吨/年）、肥田（240万吨/年）、杨柳（90万吨/年）等矿井。

电力：新建2×600MW凯里清江电厂、织金电厂、黔西电厂。扩建2×600MW清镇塘寨二期、鸭溪二期、安顺三期、织金二期； 2×660MW大方电厂二期；2×660MW金沙以大代小等。

　　煤炭深加工。综合市场需求、煤炭资源、水资源、环境容量、技术支持等条件，合理发展煤炭深加工产业，规划建设煤、电、油、气、化一体化的循环工业基地。发展以煤制醇醚燃料、烯烃等为代表的新型煤化工，力争在煤基合成油、煤制气等清洁能源和甲醇制烯烃等化工新材料方面实现新突破。重点实施织金煤制烯烃等项目，加快建设毕节煤炭精深加工基地。优化发展焦炭、化肥等传统煤化工及相关氯碱产业，支持遵义发展氯碱精细化工，在普定、大方、金沙等地合理布局合成氨项目。
　　铝及铝加工。实施煤电铝联营，做大做强铝工业，推动铝工业升级转型。推进贵阳清镇、开阳以及遵义等地区铝土矿高效开发和综合利用，增强铝土矿资源供给能力。有序、集约发展氧化铝，支持煤电铝加工一体化。重点发展铝产品深加工，提升产品附加值，有序推进铝加工项目建设。培育集煤电铝为一体的大型企业集团和产业集群，全面提升铝工业在国内外市场中的竞争力。着力打造以安顺-清镇-白云-遵义为重点的铝及铝加工产业带。
　　磷及磷化工。结合东部地区磷复肥产业转移，适度发展高浓度磷复肥，大力发展精细磷酸盐，加快黄磷尾气、磷渣和磷石膏的综合利用，推进磷矿伴生资源深度开发利用。推进开阳、息烽、瓮安、福泉、织金等磷矿资源综合利用示范基地和磷化工基地建设，加快瓮（安）福（泉）煤电磷一体化发展。
　　钢铁及铁合金。加快贵阳城市钢厂环保搬迁，优化调整产品结构，重点开发、生产特种钢及制品。开发高纯硅铁等高纯铁合金产品，实施遵义铁合金异地技改项目和红花岗坪桥铁合金升级技改项目。提升都匀、凯里铁合金精炼技术水平。

　第十一节　装备制造业

充分依托国防科技工业基地的人才、技术优势和产业基础，坚持自主创新与技术引进相结合，依托重大项目，提升装备制造业整体水平，建设西部地区装备制造业基地。
　　能矿装备。围绕国家能源资源深加工基地和重大基础设施建设需求，以装备主机为龙头，专业化协作配套为支撑，加强智能化工程机械、关键材料及精密制造技术攻关，提升成套设备生产和配套能力。重点发展液压挖掘机、特种工程机械、大中型破碎机械等专用装备，以及液压支架、采煤机、大型带式输送机等矿山机械。建立贵阳、遵义、毕节能矿装备研发基地。建设黔西煤机园区，平坝石油打捞、矿山救生设备产业园区，遵义超临界萃取装置、叶轮压气式矿石浮选设备等专业装备生产基地，都匀铸锻基地和长顺重型机械设备基地。
　　航空航天。依托安顺民用航空国家高技术产业基地、贵阳国家高新技术产业开发区，以民用新型航空产品的研制、开发为重点，着力突破通用飞机关键技术，提升航空发动机、飞机零部件等民用航空系列产品的研发能力。以飞机整机制造为龙头，重点发展通用飞机、高级教练机、民用无人机、飞机发动机、航空机载设备、航空零部件和机电一体化技术装备。加快发展航空维修、航空物流、航空会展、民航培训、民航设施制造等新型航空产业。依托航天工业基础，大力发展以航天高新技术产品为重点的航天装备产业，建设遵义航天高新技术工业园区。
　　汽车及零部件。推进资源整合和企业重组，支持发展贵阳客车、特种车，毕节重型卡车，遵义微型车、特种车，安顺轿车、客车，凯里客车，都匀矿山专用汽车等。围绕整车生产基地发展零部件产业，发展壮大以发动机、车桥、变速箱、轮胎等为重点的汽车零部件产业集群。积极发展新能源汽车。
　　特色装备。推进电子信息技术与制造技术相互融合，大力发展技术含量高、竞争能力强的特色装备制造业。贵阳重点发展磨料磨具、液压元件、成套液压系统、数控设备、电力装备等。遵义重点发展电工电器、新型化学物理电源、精密仪器、机电一体化装备和石油、核电、铝工业成套设备、新型农机装备等。安顺重点发展环保机械、现代农业装备等。都匀、凯里重点发展电梯、电子通信设备、精密数控机床等。毕节重点发展农用机械、工程机械、高压电瓷等。

　第十二节　战略性新兴产业

立足贵州资源优势和技术基础，加强政策支持和规划引导，积极推进自主创新和合作创新，尽快形成产业竞争新优势，打造西部地区战略性新兴产业基地。
　　新材料。加快建设贵阳、遵义新材料基地，改造提升金属及合金材料、化工材料生产水平，做大做强以海绵钛、钛合金、高性能铝合金、铝镁合金、高纯电解锰等产品为重点的优势新材料，推进遵义钛业及其精深加工项目，建设红花岗钛镁新材料工业园。加快发展新型复合材料及应用、新能源材料及制品、先进陶瓷材料、光电子信息功能材料等，建设息烽-乌江铝镁新材料工业园区。支持安顺发展民用航空相关新材料。加强废渣资源转化运用，提升水泥生产技术水平。加快发展以节能、环保、综合利用为重点的新型建材，大力发展资源加工废弃物为原料的新型墙体材料，实施清镇、普定粉煤灰综合利用项目。支持凯里发展玻璃深加工产业。
　　节能环保产业。加快污染物治理适用技术研发和产业化应用，发展水污染和空气污染防治设备、固体废弃物处理设备、危险废弃物无害化处置设备、环境监测设备及生物环保材料与药剂等。优先开发高低压电器设备、节能变压器、电机变频调速、路灯节能控制、半导体照明、节能泵等节能技术和产品。发展节能及环保服务业。
　　新能源。优先发展沼气、非粮生物乙醇和生物柴油等生物质能。大力发展风能，推进实施一批风力发电项目。推广应用地源热泵技术，扩展地热应用领域。积极发展太阳能光伏发电。加大新型高效电池技术、垃圾发电、光伏发电和大规模储能技术研发和应用力度，建设遵义薄膜太阳能电池设备研发生产基地。开展核能清洁能源应用研究，适时启动前期工作。
　　电子及信息技术产业。加快建设以贵阳、遵义为核心的电子与信息技术产业集聚区。贵阳重点实施乌当电子元器件生产线技术改造，发展家用视听、通信终端等消费类电子设备、新型电子元器件等产业。以贵阳数字内容产业园为基础，整合软件及系统集成企业，推进云计算研发应用，大力发展下一代互联网和物联网技术产业。遵义重点推进与重庆电子信息产业领域的配套协作，着力打造信息技术产业基地，承接计算机部件与配套产品生产、嵌入式系统、软件开发等产业。安顺以黔中新兴产业示范园区为载体，加快推进电子信息产品制造、软件及信息服务、信息化应用等产业项目建设，推动中国移动全国数据中心基地项目建设。凯里重点发展新型电子元器件、通信终端等产品，建设国内中小型精密开关基地。

　第十三节　特色轻工业

立足区域资源优势，积极构建特色鲜明、优势突出、品牌美誉度高的轻工产业体系，建设国家重要的名优白酒基地、绿色食品加工基地和民族医药基地。
　　白酒酿造。实施品牌带动战略，加强技术创新，挖掘发展潜能，增强发展后劲，创建并全力打造“贵州白酒”品牌，提升贵州白酒的综合实力和整体竞争力。稳步扩大茅台酒生产规模，确保产品质量，维护品牌声誉，发挥仁怀国酒文化特色，建设“白酒之都”。鼓励申报“酱香型白酒”地理标志，切实加强原产地保护。支持习酒、珍酒、董酒、鸭溪窖酒、金沙回沙等名优品牌白酒改造升级。支持通过收购联合重组等方式，恢复和振兴安酒、毕节大曲、匀酒、平坝窖酒等老牌名酒。建设以仁怀为核心的国家优质酱香型白酒产业带。支持都匀、凯里等发展具有民族风情的米酒、果酒产业。
　　特色食品。挖掘特色农林产品增值潜力，构建具有地方特色的农林产品精深加工体系。重点发展辣椒、马铃薯、调味品和肉制品等特色食品，积极开发无公害食品、绿色食品和有机食品，培育一批知名食品品牌。规划建设惠水-长顺、西秀-镇宁特色食品产业园区。努力提高茶叶加工能力和水平，整合品牌资源，提升都匀毛尖等黔茶知名度和市场竞争力。发展多种衍生茶产品深加工，加强茶叶原产地认证。
　　民族医药。充分发挥国家中药现代化科技产业（贵州）基地载体作用，建设中药创新药研究中心，打造国家级重点实验室和工程中心。着力打造“黔药”、“苗药”品牌和中药驰名商标，培育中药材及名牌中成药骨干企业。建设以贵阳为核心，安顺、毕节、凯里、都匀和龙里为重点的民族医药产业聚集区。加快贵阳修文、乌当、清镇，遵义深溪、董公寺，毕节大方，凯里和安顺现代化药业产业园区建设。加快GAP规范化中药材种植基地建设，重点支持发展天麻、杜仲、灵芝、太子参、金银花等特色中药材。支持贵阳实施“推动医药产业跨越千亿元行动计划”。

　第十四节　循环经济

以循环经济统领产业生态化转型，推进减量化、再利用、资源化，提高资源利用效率，培育循环经济产业体系。继续推进贵阳国家循环经济试点城市、低碳试点城市建设和节能减排财政政策综合示范工作。鼓励福泉创建循环经济示范城市。鼓励贵阳、遵义经济技术开发区等开展循环化改造。鼓励七星关区、开阳县、仁怀市、遵义县、红花岗区、绥阳县、普定县等建设循环经济型区县。着力打造清镇-黔西-织金煤电铝、煤电化循环经济示范基地。鼓励工业聚集区、产业园区及城镇实施热电联产集中供热。支持织金、金沙、大方、绥阳、普定、平坝等建设一批利用余热、余压、煤层气以及煤矸石等低热值燃料的并网发电项目。支持贵州茅台酒股份有限公司、瓮福（集团）有限责任公司开展国家资源节约型、环境友好型企业试点创建。鼓励企业进行废物交换利用、能量梯级利用、水分类利用和循环使用。支持建设一批循环经济型企业以及仁怀酒糟综合循环利用项目。开展再生资源利用和再制造，构建再生资源回收网络。

　第五章　现代服务业

全面提升服务业发展质量和水平，做大做强文化产业，提高旅游业层次，培育发展商贸物流和金融业，推动服务业大发展，构建现代服务业体系。

　第十五节　文化产业

充分发挥文化资源优势，积极引导产业集聚发展，形成多民族文化与丰富多彩的历史文化、生态文化相结合的产业格局，努力把文化产业打造成支柱性产业。加大对多民族特色文化产业发展支持力度，积极扶持民族歌舞、戏剧、服饰、民间工艺等产业化发展。充分利用民族节庆平台，大力发展节庆经济。发展壮大出版发行、影视制作、印刷、广告、演艺、娱乐、会展等传统文化产业。加快发展文化创意、数字出版、移动多媒体、动漫游戏等新兴文化产业。促进多民族文化与传统文化、新兴文化产业融合发展，加快培育特色文化产业集群。以“多彩贵州”品牌为龙头，实施“百佳文化品牌”培育工程，培育一批民族特色文化品牌。实施文化走出去工程，扩大民族特色文化在国内外的品牌影响。支持举办中国（贵州）少数民族文化产业交易博览会和亚洲青年动漫大赛，办好多彩贵州·中国原生态国际摄影大展、长征文化节等重大文化会展赛事活动。

专栏2：文化产业集聚区

贵阳：重点发展生态文化、阳明文化等，建设多彩贵州品牌研发基地，打造集多门类文化产业于一体的文化产业核心区。

遵义：重点发展红色文化、酒茶文化、沙滩文化、诗乡文化等。 安顺：重点发展屯堡文化、夜郎文化、生态文化与古生物文化等。 毕节：重点发展彝族文化、土司文化、喀斯特生态文化等，构建奢香古驿文化产业带。建设七星关-大方、黔西-织金文化产业集聚区。 都匀、凯里：重点发展苗族、布依族、侗族等民族文化，建设民族特色文化与自然山水相融合的产业集聚区。

　第十六节　旅游业

着力提高旅游业发展质量，打造具有贵州特色的旅游产业体系，努力建成国际知名、国内一流的旅游目的地和休闲度假胜地。科学规划和布局风景名胜区等景区景点，精心设计旅游线路，打造精品旅游景区。按照国际化旅游城市标准，完善旅游城市服务体系建设，重点加快建设旅游目的地营销体系和“爽爽贵阳”游客综合服务中心等重点旅游项目。加强景区内、景区与中心城镇相连的基础设施建设，全面提升旅游接待能力。积极发展原生态、自然风光、拓展体验、康体养生、休闲度假、农业观光、森林游憩等特色旅游业，打造旅游产业集聚区。促进文化和旅游融合发展，增加旅游业文化内涵，建设一批民族文化旅游中心城市、国家级风景名胜区、大型民族文化旅游景区和特色乡镇村寨，重点加强遵义、青岩、茅台等历史文化名城（名镇）以及旅游资源富集城镇保护和建设。着力搜集、整理、挖掘民族民间特色旅游商品传统工艺技术，大力开发蜡染、服饰、银饰、苗绣、漆器等具有地方和民族特色的旅游商品，推进旅游商品规模化、系列化、品牌化。高标准规划建设都匀、凯里等民族旅游商品产业园区、遵义等红色旅游商品产业园区，建设贵阳旅游商品集散中心。培育“爽爽贵阳”城市旅游品牌，整合做强黔中旅游经济区板块，实现区域旅游市场一体化，建立黔中无障碍旅游区。

专栏3：旅游业精品线路和重点景区

精品线路：贵阳-天龙-龙宫-黄果树-天星桥喀斯特自然山水和屯堡文化旅游线，贵阳-息烽-遵义-仁怀-习水-赤水红色、国酒、生态、丹霞文化旅游线，遵义会议会址-娄山关-宽阔水、双河洞-温泉-沙滩红色历史文化和生态科考旅游线，贵阳-龙里猴子沟-金海雪山-都匀斗篷山-凯里生态、民族风情旅游线，贵阳-织金洞-百里杜鹃-毕节喀斯特、高原山地运动体验旅游线。 重点景区：大黄果树景区，遵义会址景区，茅台酒文化度假区，多彩贵州城，清镇休闲体育度假区，开阳南江大峡谷景区，夜郎湖中心景区，百里杜鹃景区，织金洞景区，宽阔水、双河洞生态旅游度假区，凯里民族民间竞技赛会中心和银饰博览园等。

　第十七节　商贸物流业

围绕重要交通通道、枢纽和节点，建设一批物资集散中心、物流中心和专业市场，大力发展统一配送、连锁经营、网络购物等新兴业态，完善现代物流服务网络体系，构建区域一体化的物流新格局。大力培育和引进第三方物流企业。支持贵阳建设物流区域性节点城市，培育发展一批地区性物流节点城市。鼓励贵阳、安顺联动发展，促进贵阳、安顺空港物流发展，着力打造在西南地区有重要影响的内陆港和联系东盟的重要现代物流中心。鼓励遵义建设区域性现代商贸物流枢纽，形成黔北区域生产性服务业聚集区。支持毕节发展以能矿资源产品为主的物流产业，打造川滇黔三省结合部的重要物流中心。推动都匀、凯里重点发展以特色农产品为主的现代物流，形成西南地区连接华南地区的重要现代商贸物流枢纽。

专栏4：物流业发展基地

重点物流园区：贵阳改貌、修文扎佐、清镇，遵义南宫山和新舟空港、仁怀，安顺黄桶-幺铺，都匀，凯里，毕节远航。

农副产品综合批发交易中心：贵阳花溪、遵义虾子、安顺镇宁大山、都匀墨冲、凯里舟溪、毕节七星关。

中药材批发市场：贵阳南明、遵义汇川、毕节大方。

酒类交易市场：贵阳、遵义。

　第十八节　金融及其他服务业

加快发展地方金融机构，组建贵州银行、茅台集团财务公司，将农村信用联社改制组建农村商业银行。实施“引银入黔”工程，吸引国内大型股份制商业银行和外资银行设立分支机构。推动村镇银行、小额贷款公司和农村资金互助社建设，开展农村金融创新改革试验。支持符合条件的企业发行债券和上市。支持民营资本为主体的创业投资基金、成长型企业股权投资基金发展，完善支持中小企业发展的金融服务体系。加快发展证券业，支持本地证券机构创新发展、做优做强，积极引进优质证券期货经营机构。加快建立地方法人保险公司，积极引进国外保险公司。拓宽保险服务领域，鼓励保险参与交通、能源、水利、市政公用及电网改造等基础设施和重点产业项目建设。大力发展信息咨询、人力资源服务、科技服务、商务服务、节能环保服务、服务外包和家政、养老、社区服务、病患陪护等服务业。支持房地产业平稳健康发展。支持开展服务业综合改革试点工作。

　第六章　新型城镇化

探索山地新型城镇化道路，加快城市发展，培育重点城镇，推进城乡一体化，建设贵州城乡统筹发展的先行示范区。

　第十九节　城市发展

按照统筹规划、合理布局、完善功能、以大带小的原则，构建科学合理的城市体系，全面强化城市集聚与辐射能力，形成中心集聚、轴线拓展的城镇空间体系。
　　贵阳。优化城区结构，提升现有城区承载能力，有序拓展城市发展空间，吸引人口、产业等要素集聚。向东与龙里相连，重点建设龙洞堡东部新城，打造双龙经济带；向西与清镇连片发展，重点建设百花湖生态新城，打造集休闲度假为一体的现代商务中心；向南至惠水、长顺方向，重点建设溪南新城，打造新型工业化走廊；向北与麦架、沙文、扎佐连片发展，重点建设修文工业新城，打造高新技术产业集群。把贵阳建设成为全国生态文明城市、西部地区高新技术产业重要基地、区域性商贸物流会展中心。 遵义。重点推进新蒲新区建设，加快高坪组团-高桥、忠庄-乌江、南关-深溪片区开发和老城改造，推动遵义县、绥阳县撤县建区，建设遵义至仁怀城际联系干道和遵义董公寺至绥阳洋川城市干道，打造区域特大城市，形成贵州与成渝经济区合作的重要门户城市。全面推进与贵阳协同发展，构建贵阳-修文-息烽-开阳-遵义为主轴的城市带。
　　安顺。统筹安顺、贵阳城市规划，促进城市功能协调发展和布局优化，推进基础设施共建共享。支持平坝县、普定县撤县建区，建设安顺特大城市。向西南带动镇宁发展，向东北与贵阳百花湖生态新城对接，向东与花溪溪南新城融合发展。重点发展旅游、航空航天、电子信息、农林产品加工、现代物流等产业，打造成为国际旅游目的地城市和区域装备制造业基地。
　　毕节。优化中心城区空间布局，加快推进城市向东扩展，支持大方县撤县建区。建设七星关至大方城际间联系干道。推动与贵阳聚合发展，构建贵阳-清镇-织金-黔西-毕节为主轴的城市带。
　　都匀、凯里。加快培育都匀、凯里区域中心城市。以麻江为节点城市，沿凯麻公路、贵新高速公路改造建设城市干道，建设凯里、下司、宣威至都匀城市主干道，推动都（匀）麻（江）凯（里）一体化发展。推进与贵阳联动发展，构筑贵阳-龙里-贵定-福泉-都（匀）麻（江）凯（里）为主轴的城市带。

　第二十节　贵安新区

　　坚持高起点规划、高标准设计、高质量建设、高效率开发，抓紧编制实施《贵安新区总体方案》，科学确定战略定位和功能分区，集中打造新体制、高科技、开放型的新兴产业集聚区和现代化、人文化、生态化的新兴城市，建成国家内陆开放型经济示范区和黔中经济区最富活力的增长极。加快市政基础设施建设，构建城际快速交通干道，建设完善的公共服务设施。大力培育现代产业体系，建设高端装备制造业基地、绿色食品生产加工基地和旅游休闲目的地、区域性商贸物流中心和科技创新中心。推进体制机制创新，加大行政管理和社会管理改革力度，赋予贵安新区深化体制机制改革和推进开放先行先试权。研究设立贵安新区建设专项资金，专项用于重大基础设施和公共服务项目建设。推动组建贵安新区投资开发公司，设立创业投资引导资金和产业投资基金，支持成长型企业发行企业债券。贵安新区建设用地指标纳入省级土地调控计划，积极开展土地流转制度改革，探索有效的土地流转方式和补偿机制。

　第二十一节　重点城镇

统筹规划城镇体系，提高城镇综合承载能力，促进城镇协调发展。大力发展县域经济，将有条件的重点中心城镇培育成小城市。按照宜农则农、宜工则工、宜商则商的原则，着力优化城镇空间布局，强化产业支撑，突出自然、历史、文化和民族特色，建设一批特色鲜明的重点镇。

专栏5：重点镇体系

交通枢纽型城镇：修文久长，仁怀大坝，平坝马场，镇宁大山，凯里三棵树，麻江碧波，大方黄泥塘，织金以那。

旅游景点型城镇：息烽温泉，遵义乌江，汇川板桥，绥阳温泉，仁怀茅台，西秀龙宫，平坝天龙，惠水羡塘，贵定盘江，瓮安龙塘，大方雨冲，黔西洪水，金沙岩孔，麻江下司。

绿色产业型城镇：开阳龙岗，普定坪上，西秀双堡，贵定云雾，龙里醒狮，麻江宣威。

工矿园区型城镇：开阳金中，息烽小寨坝，清镇站街，仁怀二合，遵义尚嵇，绥阳风华，西秀宋旗，平坝夏云，都匀大坪，福泉龙昌，贵定昌明，惠水长田，长顺威远，龙里谷脚，瓮安银盏，凯里炉山，七星关岔河，织金珠藏。 商贸集散型城镇：修文扎佐，清镇卫城，遵义虾子，普定马场，都匀王司，惠水摆金，长顺广顺，瓮安猴场，大方理化，金沙沙土，黔西钟山。

　第二十二节　市政基础设施

加快建设城镇交通基础设施。完善贵阳环线，建设遵义、安顺、毕节、都匀、凯里等区域重点城市环线，构筑城市路网骨架。加快建成贵阳城市轨道交通，规划实施联结经济区大中城市和县城轨道交通工程。加强城市综合交通枢纽设施建设。加快城镇供水设施改造与建设，提升水质监测与应急能力。实施城市河流水污染防治工程，加快城镇污水处理设施建设，新建、扩建一批城镇污水处理厂，完善配套管网，推进管网雨污分流改造，提高城市生活污水处理及污泥处理处置与资源化利用能力，加强农村生活污水处理。到2015年，各中心城区污水实现全收集、全处理，城市生活污水集中处理率达到85%以上，城市污水再生利用率达到20%以上，力争基本实现所有县和重点建制镇具备污水处理能力。统筹城乡垃圾处理与管理，提高垃圾无害化处理水平，推进垃圾处理产业化发展。到 2015 年，生活垃圾无害化处理率达到80%以上。加强给排水、供热、供气、通信、绿化等基础设施和公共服务设施建设，努力营造良好的人居环境。

专栏6：市政基础设施重点工程

轨道交通：贵阳城市轨道交通2号线（一期）工程，城市轨道交通1号线工程。城市干道：建设联结贵阳龙洞堡新城的贵阳-龙里城市干道，联结贵阳溪南新城的花溪-惠水-长顺城市干道。贵阳安顺同城化联结干道平坝-羊昌-党武、金阳-清镇-平坝-西秀-普定，平坝-马场-石板，清镇-马场-长顺城市快速干道。贵阳-乌江-红花岗忠庄，遵义-仁怀城际联系干道。遵义-新蒲-绥阳城市干道。毕节大方同城化以及都（匀）麻（江）凯（里）一体化联结线。 污水垃圾处理：新建城市污水处理设施项目20个，规模33.6万吨/日。乡镇污水处理设施项目140个，规模27.4万吨/日。续建城市垃圾无害化处理设施项目，规模1010吨/日。新建乡镇垃圾无害化处理设施项目67个，规模2480吨/日。

　第二十三节　城乡统筹发展

全力推进城乡规划建设一体化，建立覆盖城乡的规划体系。统一规划基本农田保护区、居民生活区、工业园区、商贸区、休闲区、生态涵养区等，使城乡发展互相衔接、互相促进。推动城市基础设施向农村延伸，提高城乡基础设施一体化水平，实施水、电、路、气、房和优美环境六到农家工程。建立和完善覆盖城乡的基本公共服务保障机制，把社会事业建设重点放在农村，逐步缩小城乡公共事业发展差距。健全城乡统一规范灵活的人力资源市场，逐步形成城乡平等就业的环境。积极稳妥推进户籍管理制度改革，引导有稳定劳动关系并在城镇居住一定年限的农民工在城镇落户，保障农民工在城镇逐步平等享受市民权益。加强农民工权益保护制度建设，在农民工流动较为集中的地区，依托现有公共服务设施建设一批农民工综合服务中心。推进贵阳、遵义统筹城乡综合配套改革试点。

　第七章　基础设施

加强以交通、水利为重点的基础设施建设，加快形成适度超前、设施先进、功能完善、覆盖城乡、畅通便捷、安全高效的基础设施网络，增强区域发展支撑能力。

　第二十四节　交通运输

推进各种运输方式统筹协调发展，形成连接南北、沟通东西的交通网络格局，构建高效便捷的现代综合交通运输体系。 铁路。加快建设贵广铁路、长昆客运专线，开工建设成贵铁路、渝黔铁路扩能工程，尽快贯通隆昌至黄桶铁路，规划研究黄桶至百色等铁路，逐步构成连接西南、珠三角等地区的大能力通道。研究建设黔中经济区城际轨道交通及部分支、专线铁路，形成较为完善的铁路交通网络。继续实施铁路电气化改造，建设和改造贵阳、毕节等铁路枢纽，推进货运枢纽建设。
　　公路。加快国家高速公路建设和国省干线公路升级改造，进一步优化路网结构，提高通达能力。加大以经济路、旅游路、连接中心城市的跨区域公路及过境公路、重点乡镇、重点工矿区与高速公路的联络线为重点的路网改造力度。加强农村公路建设，继续实施农村公路通达通畅工程，完善路网体系。加强县（市、区）中心客运站和农村客运站场的建设和改造。
　　水运。改善内河航运条件，完善航运配套设施，提升航道等级，重点建设乌江（乌江渡-龚滩）航道，加快推进枢纽通航建筑物建设，形成“北入长江”通航500吨级船舶的四级航道。开展赤水河白杨坪-岔角航道整治前期工作。启动乌江沿江港口扩能工程，加快建设开阳港、洛旺河、息烽港、大塘口、瓮安港、江界河、遵义港、楠木渡、乌江渡等重点港区。继续大力实施库区航运和农村渡口改造工程。
　　民航。推进贵阳龙洞堡国际机场建设，适时实施三期扩建工程，配套完善相关服务设施，研究开辟中远程国际航线。加快推进遵义机场建设，新建毕节、仁怀机场，改扩建安顺机场。积极发展通用航空，适时试点开放低空空域。

专栏7：交通建设重点工程

铁路：建设贵阳北站以及毕节至织金、林歹至织金铁路，规划研究黄桶至百色、叙永至毕节、昭通至黔江铁路贵州段、黔桂铁路增建二线等铁路项目。规划研究建设黔中经济区城际轨道交通。

高速公路：建设大兴-思南-遵义-毕节-都格、清镇-织金-纳雍、毕节-生机高速公路。改扩建贵新高速公路贵阳至麻江段。

　第二十五节　水利设施

统筹考虑经济社会发展对水资源的需求，抓紧实施《贵州省水利建设生态建设石漠化治理综合规划》中的大中小型水资源工程，基本解决工程性缺水问题。加快建设黔中水利枢纽一期工程，积极推进夹岩等大中型水源工程建设。完善金沙胜天、织金大新桥、西秀油菜河、福泉高车等“滋黔”一期工程配套渠系设施，加大实施烟水配套和瓮福、金黔、遵义、安西等大中型灌区续建配套与节水改造工程力度，推进小水窖、小塘坝、小堰闸、小泵站、小渠道等“五小”水利工程建设。统筹利用地表水和地下水资源，加强岩溶地下水和地下暗河开发利用，建设一批应急水源工程，全面提高区域抗旱应急能力。加强城市、重点城镇和工业园区供水管网建设，保障城镇用水安全。加快民生水利建设，全面解决农村人口的饮水安全问题。加快重点城镇和河道的防洪治理，积极推进重点中小河流治理、病险水库除险加固和山洪地质灾害防治，全面提高区域防灾减灾能力。建立健全差别水价制度，开展水权交易试点。

　第二十六节　能源通道

加快建设连接能源基地与主要负荷中心的骨干坚强智能电网，形成500千伏西电东送输电通道和贵州省内“三横两中心”网格型电网格局。加强电网智能化建设和运营管理，增强承接各类型电源分区分层接入能力。提高电网建设标准，增强跨区域送电的可靠性和抗御自然灾害能力。实施新一轮农村电网改造升级工程，加大农村电网、工业园区电网和配电设备建设力度，提升并统一城乡电网建设技术标准，增强供电能力。大力发展带式输煤，加快既有输煤线扩能改造，推进盘县-安顺大型带式输煤工程建设。积极开展管道输煤前期工作。改造提升电煤运输公路等级，启动建设一批连接煤矿与重化工业基地的铁路运煤专线，完善矿区与铁路货运场站和水运码头联络通道。依托中缅天然气管道、中卫至贵阳天然气管道，加快建设黔中经济区节点城市支线管网。支持建设成品油储备设施。

专栏8：“三横两中心”电网项目

“三横”：毕节-鸭溪-遵义东-大兴组成的北通道，安顺-贵阳西-贵阳-福泉-铜仁组成的中通道，兴仁换流站-独山组成的南通道。 “两中心”：以贵阳和遵义为中心500千伏环网供电系统。

　第二十七节　信息

加快推进电信网、广播电视网和互联网“三网融合”。推进统一经济区内电话区号和移动网络，构建无障碍信息网络平台。加强农村通信、广播电视等基础设施建设，扩大广播电视和互联网在农村的覆盖面，实现数字电视、宽带进村入户。加强信息服务能力建设，加快电子政务工程建设，实施农村信息化建设与服务工程，强化信息网络安全与应急保障基础设施建设，着力提升电子政务、电子商务、地理信息、远程教育、远程医疗等服务能力，提升区内经济社会运行的智能化程度。加强邮政普遍服务体系建设，开展邮政、电信普遍服务补偿机制试点。

　第八章　农业与生态环境

依托山地立体气候和生态环境良好特点，积极发展名特优新农产品，建设生态农业基地。强化生态建设，加强生态保护，构筑区域经济社会发展生态安全屏障。

　第二十八节　特色优势农业

提高农业综合生产能力，大力发展特色农业，实施山地高效立体农业工程，建设贵阳、遵义、毕节等山区现代农业示范区和黔东南、黔南生态农业示范区、安顺山地农业机械化示范区等现代农业产业园区，走规模适度、加工精细、品质优良、特色鲜明的特色农业发展道路。加大特色农产品注册商标和地理标志开发保护力度，打造一批具有较强影响力的品牌。
　　稳定发展粮食生产，以优质水稻、专用玉米、优质油料、马铃薯等为重点，实施商品粮油高产示范工程，建设一批区域性商品粮油基地，增强粮油保障能力。支持仁怀、金沙建设优质酿酒专用高粱基地。大力发展反季节蔬菜和无公害蔬菜，建设面向泛珠三角区域的蔬菜生产基地，加快建设龙里至贵定湾滩河流域蔬菜产业园区。支持辣椒产业发展，重点建设遵义、花溪、大方、平坝、龙里、清镇等辣椒基地。大力发展食用菌生产，重点建设织金、镇宁有机竹荪生产基地。积极发展名特优果，建设以核桃、板栗、黄梨、金秋梨、刺梨、杨梅、猕猴桃、柑橘、蓝莓等为代表的传统地方优质水果、干果基地。以平坝县、开阳县、都匀市、贵定县、遵义县、七星关区、金沙县、大方县为重点，加快建设高档名优生态绿茶基地。积极选育烤烟新品种，提升烤烟单产和质量，建设毕节、遵义等国家优质烟叶生产科技示范基地。建设观赏花卉和鲜切花基地，发展壮大花卉产业。稳步发展生态畜牧业和淡水养殖业。支持生猪生产优势县发展，加强优质猪生产基地县建设，提高出栏率和商品率。稳步发展壮大以肉羊为主的草地生态畜牧业，建设仁怀黔北麻羊原种繁育中心，在条件具备的地区建设商品羊生产基地。积极发展附加值高的鲟鱼、鲑鳟鱼等特色生态养殖。

　第二十九节　生态综合治理

坚持治水与固土并重，生物措施、工程措施和技术措施并举，整合各类专项资金，加大石漠化综合治理力度。进一步推进天然林资源保护、长江和珠江流域防护林体系、速生丰产林、水土保持、岩溶地区草地治理等重点生态工程建设。巩固发展退耕还林成果，对生态位置重要的陡坡耕地逐步实施退耕还林还草。加强水土流失综合防治，加快坡耕地综合整治。组织实施易地扶贫搬迁工程。加大湿地保护力度，维护湿地生态系统的健康和安全。加强自然保护区、森林公园、湿地公园、风景名胜区保护和建设，保护生物多样性，提升生态系统功能。建立和完善生态补偿机制，鼓励在珠江流域率先开展生态补偿。大力发展林下经济，积极推进山区特色经济林、工业原料林建设。深化集体林权制度改革。加强农村能源建设，推广农村节能技术，优化农村能源结构。深入推进贵阳全国生态文明城市建设，扩大生态文明贵阳会议影响，建设一批全国生态文明示范县。

　第三十节　加强环境保护

严格水功能区管理，核定水域纳污容量，切实推进乌江和赤水河中上游、珠江上游水污染防治，严格控制入江污染物排放量。加强水源地保护，加大百花湖、红枫湖、夜郎湖、阿哈水库、花溪水库、松柏山水库和水泊渡水库等重要水源涵养区、集中式饮用水源地环境治理和保护力度。加强对电力、钢铁、有色、建材、化工等重点行业的二氧化硫、氮氧化物排放以及工业烟尘、粉尘和城市扬尘的污染控制。加大尾矿库和重金属污染治理力度。加快淘汰落后产能，支持重点行业实施技术改造和清洁生产，支持高载能行业建设一批年节能 5000 吨标准煤以上的重点节能项目。全面加强矿区生态保护和环境综合治理，完善矿山环境治理恢复保证金制度。积极开展节水型城市和节水示范县建设，实施建筑节能和公共机构节能示范工程。加强农村环境综合整治，实施农业生产清洁工程、农村生活环境净化工程、农林剩余物综合利用工程，推动乡镇和农村污水、垃圾收集处理设施建设，改善村容村貌。加强农业面源污染防治关键技术的科技攻关，大力推广测土配方施肥技术和生物农药。加快推进规模化畜禽养殖场和养殖小区污水和固体废弃物处理设施建设。加强对高浓度、高风险、重金属污染土壤的修复治理和农业投入品废弃物回收利用。完善环境监测预警系统，建立环境污染事故应急处置体系。

　第九章　内陆开放高地

坚持解放思想，扩大开放，深化区域合作，积极承接产业转移，大力发展内陆开放型经济，构建全方位、多层次、宽领域的对内对外开放新格局。

　第三十一节　对内对外开放

充分利用中国-东盟自由贸易区、大湄公河次区域、泛珠三角地区等平台，加强与东南亚、南亚等国际区域在经贸、教育、科技和文化等领域合作，承接产业转移，打造东西合作示范基地。深化与成渝经济区、长株潭城市群、北部湾经济区等周边区域的互动合作，积极推进基础设施互联互通和生产要素自由流动，全方位加强在能源、旅游、生态、产业等领域的协作，实现优势互补。强化与贵州省内其他区域的合作，打破行政界限和市场分割，加快基础设施、公共服务和社会管理等方面的对接与整合，提高区域发展一体化水平。

　第三十二节　完善基础平台

按照整体规划、严格管理、滚动发展、务求实效的原则，发挥产业园区的集聚、引领和辐射作用，促进内陆开放型经济发展。支持贵阳、遵义经济技术开发区和贵阳高新技术产业开发区扩区调位、一区多园建设。支持贵阳白云、遵义（苟江、和平）、安顺、都匀、凯里、仁怀等省级园区建设和发展，高标准规划建设毕节等一批省级产业园区。支持在有条件的地区设立高新技术开发区，建立工程研发中心、高端人才创业园，吸引国内外有实力的企业集团投资创业。支持省级重点工业园区积极创建国家新型工业化产业示范基地。支持在条件成熟的地区设立海关特殊监管区域，完善政策与功能，鼓励加工贸易企业向海关特殊监管区域集中。加强贵阳、都匀、凯里出口基地和输港澳鲜活产品出口基地建设。办好中国（贵州）国际酒类博览会。

　第三十三节　承接产业转移

改善投资环境，增强招商引资吸引力，完善招商引资项目库，积极争取世界500强、国内500强、民营企业500强和中央企业直接投资。建立招商引资重大项目协调机制，建立和完善招商引资考评、奖惩目标责任制。推进贵州·江苏信息产业园建设，实现省、市、县共建，加快构建东西合作互动产业示范园区。着力建设以发展磷化工、冶金、机电制造为主的息烽-乌江-汇川产业承接园区，以发展纺织、服装、玩具、家电为主的都匀-麻江-凯里承接园区，以发展能矿机械制造、中药材精深加工、特色食品为主的黔西-大方-七星关产业承接园区，以发展轮胎、生物医药、飞机发动机为主的贵阳-安顺产业承接园区。

　第十章　社会事业

积极发展教育、科技、文化、卫生等各项社会事业，完善社会保障体系，全面提升基本公共服务能力，推动和谐社会建设。

　第三十四节　教育

优化教育资源配置，加大公共教育资源投入力度。加快普及学前教育，实施农村学前教育推进工程，每个乡镇和具备条件的县城社区建设一所公办幼儿园，学前三年教育毛入园率达到70%。巩固和提高义务教育水平，科学规划、合理布局农村中小学校，加强城镇中小学校和县镇薄弱学校建设，推进义务教育学校标准化建设。加大农村寄宿制学校建设力度，力争全面解决农村初中寄宿生住宿问题，适度发展寄宿小学，推进义务教育均衡发展。加快普及高中阶段教育，实施民族地区教育基础薄弱县普通高中建设工程，着力改善办学条件，力争高中阶段毛入学率达到80%以上。以农村中等职业教育为重点，创建一批示范性中等职业学校，鼓励建设茅台职业技术学院。加快贵州大学、贵州民族大学校区扩建工程，推进贵阳花溪和遵义新蒲高校聚集区建设。制定并完成贵州省“十二五”高等学校设置规划，支持建设贵州理工学院等高校，研究组建贵州民族医药学院和遵义大学等高等院校。

专栏9：教育重点工程

实施农村义务教育薄弱学校改造计划、中西部农村初中校舍改造工程、义务教育学校标准化建设工程、中等职业教育基础能力建设工程、幼儿园建设工程、特殊教育建设工程、普通高中建设工程、高等教育学科建设工程、管理和教师队伍素质提高工程等九大教育重点工程。

　第三十五节　科技

整合创新资源，聚集创新要素，培育创新人才，强化创新功能，发挥贵阳、遵义建设创新型城市示范带动作用，建设具有核心竞争力的创新型区域。加快国家地方联合平台建设，重点推进精密铸造、植物分子育种、干细胞应用技术、智能计算与控制技术工程、陆地交通灾害防治工程等省级工程研究中心和工程实验室建设成为国家地方联合平台。组织实施若干国家重大产业创新发展工程。支持有条件的企业组建技术中心。鼓励企业与高校、科研院所共建创新研发平台。推进建立工程技术研究院。积极推动技术创新，实施重大科技计划，力争在能源、民族制药、烟酒、磷煤化工、新材料、信息技术、生物技术、航空航天、节能环保等领域取得重大突破，形成一批具有自主知识产权的核心知识和技术。加快建设科技咨询、成果转化、技术交易等科技中介服务机构，构建技术服务平台。

　第三十六节　公共文化

积极发展公益性文化事业，加快实施贵州省博物馆、遵义会议会址等重点文物保护单位保护和重大文化设施建设工程，加强市（州）图书馆、文化馆、博物馆建设，加快推进县（市、区）图书馆和文化馆、乡镇综合文化站、村（社区）文化室建设，进一步推进公共文化机构免费开放。实施文化信息资源共享、广播电视“村村通”、农村电影放映、农家书屋等文化惠民工程，建设一批公共电子阅览室，推进广播电视直播卫星公共服务工作，建立覆盖城乡的公共文化服务体系。实施优秀文化遗产保护工程，加强红色文化遗址、重点文物保护单位、非物质文化遗产、历史文化名城名镇名村的保护和传承。实施对外文化交流精品战略，开展以民族原生态文化、文物、杂技、民间工艺、民族民间歌舞等为代表的精品项目文化交流。加强现有公共文化服务设施的管理，提高使用效率。加快建设现代传播体系。加强城乡体育健身场地和设施建设，开展特色体育休闲运动，发展竞技体育、大众体育、民族体育，促进全民健身。

　第三十七节　医疗卫生

积极推进医药卫生体制改革，以逐步实现人人享有基本医疗卫生服务为重点，建设覆盖城乡的公共卫生服务体系、医疗卫生服务体系、医疗保障体系和药品供应保障体系，形成四位一体的基本医疗卫生制度。加强市州级医院、基层医疗卫生机构能力建设，推进全科医生临床培养基地建设。完善农村急救、卫生监督、重大疾病防控等公共卫生服务网络，抓好重点传染病、慢性病、职业病、地方病和精神疾病防治工作。抓好妇幼保健工作，提高住院分娩率，降低孕产妇死亡率、婴儿死亡率和五岁以下儿童死亡率。加强人口和计划生育工作，完善人口和计划生育服务体系，稳定低生育水平。巩固和完善新型农村合作医疗制度，实施基本药物制度，促进基本公共卫生服务均等化。推进公立医院改革，鼓励社会资本以多种形式举办发展医疗机构。

　第三十八节　就业和社会保障

千方百计促进和扩大就业。实施劳动力素质提升工程，提高职业技能和综合素质。大力发展劳动密集型产业、服务业和小型微型企业，多渠道开发就业岗位。加快实施农民创业促进工程，以创业带动就业。加强对就业困难群体的就业援助。完善就业权益保障制度、劳动关系协调机制和劳动争议处理制度，保障劳动者合法权益。加快建立城乡统筹的社会保障体系，提高保障水平，率先实现区域内社会保障统筹。完善企业职工基本养老保险制度，城镇居民社会养老保险制度和新型农村社会养老保险制度。推动城镇职工、城镇居民基本医疗保险和新型农村合作医疗的有机衔接。完善城乡居民最低生活保障制度，逐步提高保障水平。进一步提高失业、工伤、生育保险统筹层次，完善各项社会保险关系转移接续办法。建立健全城乡社会救助体系，加快发展慈善和残疾人事业。继续加大保障性安居工程建设力度，制定吸引社会资金投资建设保障性住房的政策措施，推进廉租房建设，规范发展经济适用住房，大力发展公共租赁住房。加快农村教师和医疗卫生人员周转房建设。

专栏10：就业和社会保障重点项目

基层劳动就业和社会保障综合服务中心建设工程：建设一批县、乡镇（街道）劳动就业和社会保障服务中心，争取所有街道、乡镇都建立劳动就业和社会保障服务中心，所有社区、行政村都设立劳动就业和社会保障服务站。 社会保障一卡通：全面发行社会保障卡，积极推进社会保障卡应用。 地市级人力资源市场和社会保障服务中心建设：在地级市建设一批人力资源市场和社会保障服务中心。

　第十一章　深化改革

深化行政管理体制改革，加快要素市场体系建设，开展重点领域综合配套改革，切实加快体制机制创新，为黔中经济区发展提供强大的动力和体制保障。

　第三十九节　行政体制改革

深化省直管县（市）试点改革，扩大市、县政府经济社会管理权限，增强市、县政府提供公共服务的能力。积极推进行政审批制度改革，继续清理和调整行政审批项目和部门收费，逐步推 行审批管理“零收费”制度。优化政府组织结构，减少行政层级，理顺职责分工，提高行政效率，降低行政成本，建立“决策科学、分工合理、执行顺畅、运转高效、监督有力”的行政管理体制。研究推进下放和扩大产业园区管理权限，创新和完善产业园区管理体制。注重加强政府社会管理和公共服务职能，鼓励公民和社会组织参与社会管理。研究建立区域统一的政府绩效考核制度。合理调整行政区划。

　第四十节　发展非公有制经济

认真贯彻落实国家扶持非公有制经济的政策措施，营造良好的政策、制度环境，在财政税收、投融资、资源利用、人才政策等方面，对非公有制经济给予大力扶持。进一步放宽准入条件，鼓励和支持非公有制企业通过收购、兼并、控股参股、转让经营权等多种形式，参与国有企业改革和战略性调整。大力培育和引进信用担保机构，完善资本金补充和风险补偿机制，积极开发适应非公有制企业的信贷服务项目和信贷产品，建立和完善非公有制企业多层次融资服务体系。大力发展各类咨询、认证等社会服务机构和中介组织，规范和发展各类行业协会、商会等自律性组织，充分发挥其联系政府和非公有制经济的纽带作用。

　第四十一节　创新土地矿产资源开发利用机制

实行差别化土地政策，统筹安排建设用地，优化用地布局，贵州省土地利用指标优先保障黔中经济区合理用地需求。加大土地整理、复垦力度，加快中低产田改造和高标准基本农田建设。着力盘活闲置土地资源，科学利用荒山、荒坡，增加建设用地供给。推进农村以集体土地入股，共享工业园区开发成果的模式和方法。推进矿产资源综合利用，大力发展绿色矿业和循环经济，创新矿产资源节约技术和机制。支持开展煤炭矿业权审批改革试点，合理布局和有序投放矿业权，开展地质找矿新机制试点。

　第四十二节　创新人才开发机制

完善政府宏观管理、市场有效配置、单位自主用人、人才自主择业的人才管理体制。完善国民教育和终身教育体系，增加博士授权单位，推进贵州师范大学、贵州财经大学、贵州民族大学等建立博士授权点，设立专业学位博士点，调整硕士研究生招生结构，增加专业硕士学位研究生的招生比例。大力实施高技能人才开发工程，加大高级技工人才培养力度。以鼓励扶持区内创业和引入区外企业为重点，建立企业家人才的发现和集聚机制。建立科学化、社会化的人才评价发现机制和有利于人才脱颖而出的选拔制度，形成能岗相适、用当其时、人尽其才、才尽其用的使用机制。结合贵州实际，继续组织实施好新世纪百千万人才工程、长江学者奖励计划、“千人计划”、“西部之光”、“博士服务团”等国家级人才工程，实施“贵州专门人才培训工程”、“院士专家援黔行动计划”和“贵州高原学者”计划，选拔和引进一批带动重点学科、特色优势产业发展的领军型中青年人才。支持建设遵义干部学院。

　第十二章　规划实施

加强组织领导，落实保障措施，完善政策体系，调动各方面积极性，确保完成规划任务，实现规划目标。
　　贵州省人民政府成立黔中经济区协调领导小组，负责贯彻落实黔中经济区发展的重大决策部署，审定重要规划、重大政策措施，统筹协调重大项目和重大问题。领导小组下设办公室，负责根据规划要求研究制定规划实施意见和推进行动计划方案。开展对规划实施情况的跟踪分析和监督，组织开展规划实施中期评估。加强与各市（州）、市（县、区）人民政府的沟通协调，打破行政区划，协调推进黔中经济区发展，努力促进黔中经济区实现新跨越。
　　国务院西部地区开发领导小组成员单位及有关部门要加强对黔中经济区发展的指导、协调和支持。国家发展改革委要会同有关部门和贵州省人民政府加强对本规划实施情况的跟踪分析，并将实施情况向国务院报告。

© 北大法宝：（[www.pkulaw.com](https://www.pkulaw.com/)) 专业提供法律信息、法学知识和法律软件领域各类解决方案。北大法宝为您提供丰富的参考资料，正式引用法规条文时请与标准文本核对。
欢迎查看所有[产品和服务](https://www.pkulaw.com/helps/2.html)。法宝快讯：[如何快速找到您需要的检索结果？](https://www.pkulaw.com/helps/70.html)[法宝](https://www.pkulaw.com/helps/69.html)V6有何新特色？

扫描二维码阅读原文

**原文链接：**<https://www.pkulaw.com/chl/70a3e0f46eda0820bdfb.html>
